# Supplementary material for: A bimodal distribution of haze in Pluto’s atmosphere
Source: Nat Commun. 2022 Jan 11;13:240. doi: 10.1038/s41467-021-27811-6 (PMC8752795; doi:10.1038/s41467-021-27811-6)
Supplement: Supplementary file 1 — Supplementary Information [file 41467_2021_27811_MOESM1_ESM.pdf]

A bimodal distribution of haze in Pluto's atmosphere

Supplementary Information

Supplementary Table 1. List of MVIC observations used in this work.

MET	Time	Phase Angle (degree)	Distance (10^3km)	Resolution (km)
0299162512	2015-07-14 T06:50:12	18.2	246.1	4.87
0299178092	2015-07-14 T11:10:52	38.8	33.1	0.67
0299193157	2015-07-14 T15:20:58	169.4	175.3	3.47

Supplementary Table 2. Summary of tested scenarios.

	Distribution	Morphology	Free parameters	Goodness*
1	Monodispersed	Aggregates with varying D_f	D_f, r_m, N_m, n_a	–
2	Monodispersed	Aggregates with varying D_f & Surface	D_f, r_m, N_m, n_a	–
3	Bimodal	Aggregates with varying D_f & Spheres (D_f=3)	D_f, r_m, N_m, n_a, R_s, n_s	8.6
4	Bimodal	Spheres (D_f=3) & Spheres (D_f=3)	R_{s1}, n_{s1}, R_{s2}, n_{s2}	25.1
5	Log-normal	Spheres (D_f=3)	μ, σ, n_0	76.0
6	Power-law	Spheres (D_f=3)	p, n_0	67.4
7	Exponential	Spheres (D_f=3)	α, n_0	125.6
8	Log-normal	Aggregates with D_f=2	μ, σ, n_0	17.9
9	Power-law	Aggregates with D_f=2	p, n_0	21.7
10	Exponential	Aggregates with D_f=2	α, n_0	19.3

*Note: Goodness is defined as the mean value of -ln(p), where p is the posterior probability shown in Equation (20). The values of the monodispersed distributions are not shown, as only forward scattering observations were considered under these scenarios.

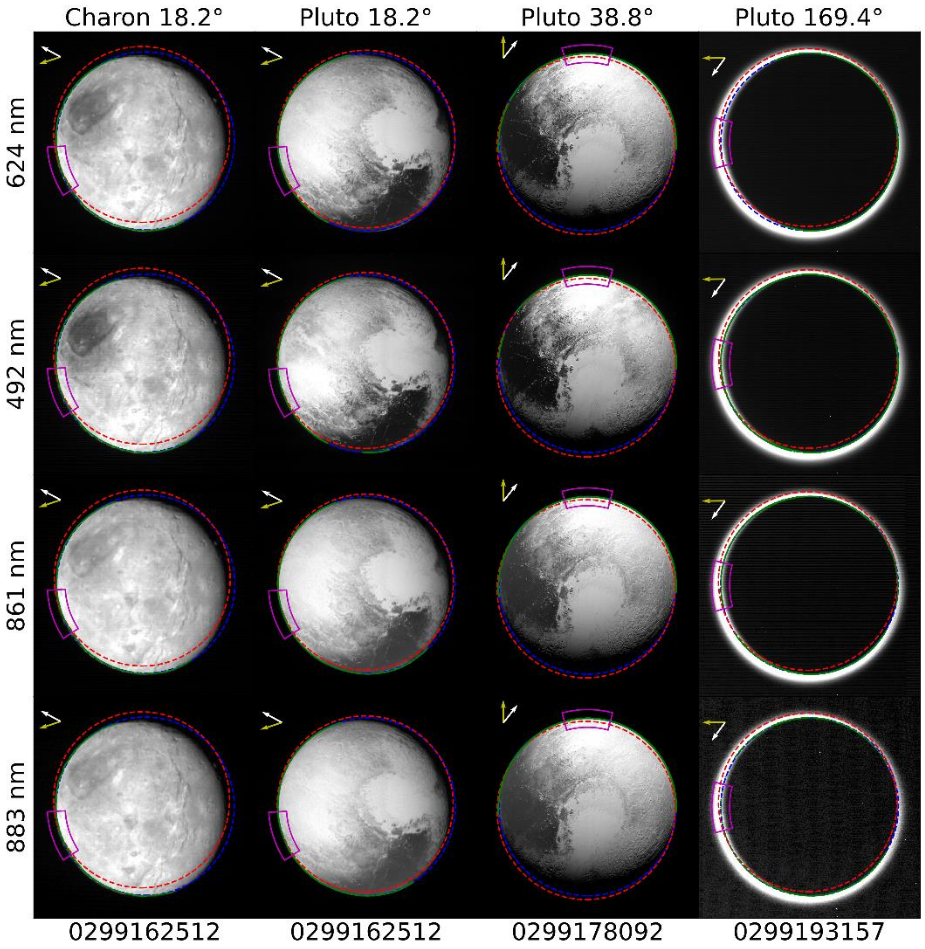

Supplementary Figure 1. MVIC observations of Charon and Pluto. The images are taken at mission elapsed time (MET) of 0299162512, 0299178092, and 0299193157. Images at different wavelength channels are presented in each row, while the columns are for different targets and phase angles. Red and blue dashed circles denote the prediction of target locations using navigation data and those derived by fitting the edge of targets using the green dots, respectively. The magenta curved boxes show the regions which are used for data analysis, presented in Supplementary Figure 2. The yellow and white arrows denote the directions of the Sun and Pluto's north, respectively.

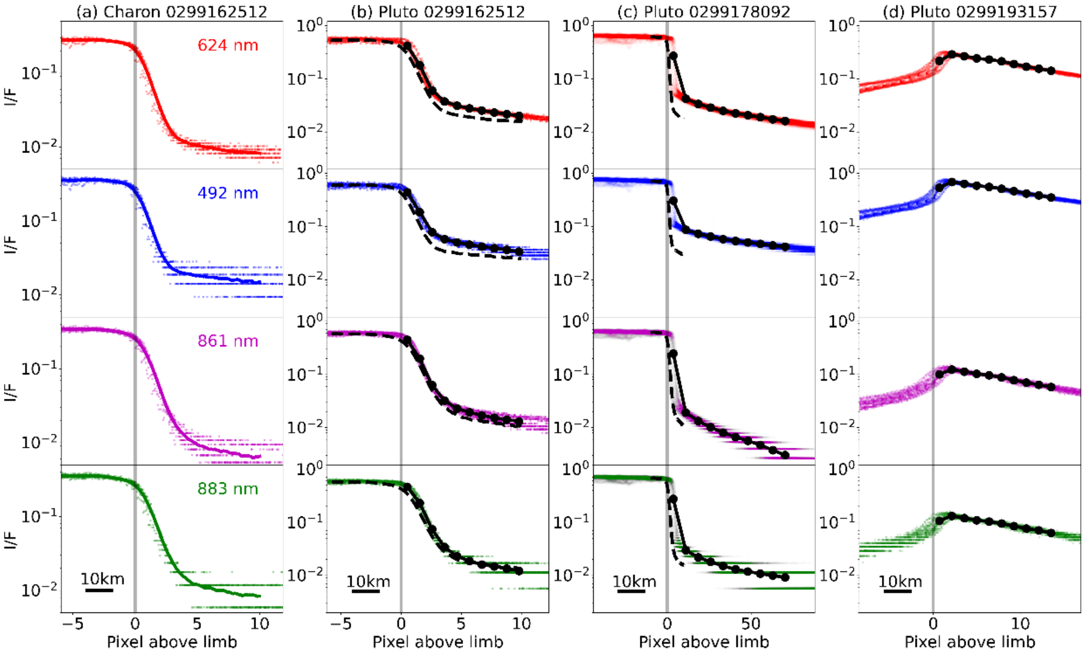

Supplementary Figure 2. I/F profiles derived using MVIC images. The profiles are plotted as a function of pixel distance above the limb. The sequence of the panels is the same as Supplementary Figure 1. Observed values are denoted as colored dots, and the vertical solid grey lines show the locations of target limbs. Moving averages of observations of Charon are shown as colored solid lines in (a), and used for stray light correction shown as black dashed lines in (b) and (c). Binned observations of Pluto are denoted as black dots and solid lines in (b)-(d). A black bar showing the scale of 10km is at the lower left corner of each column.

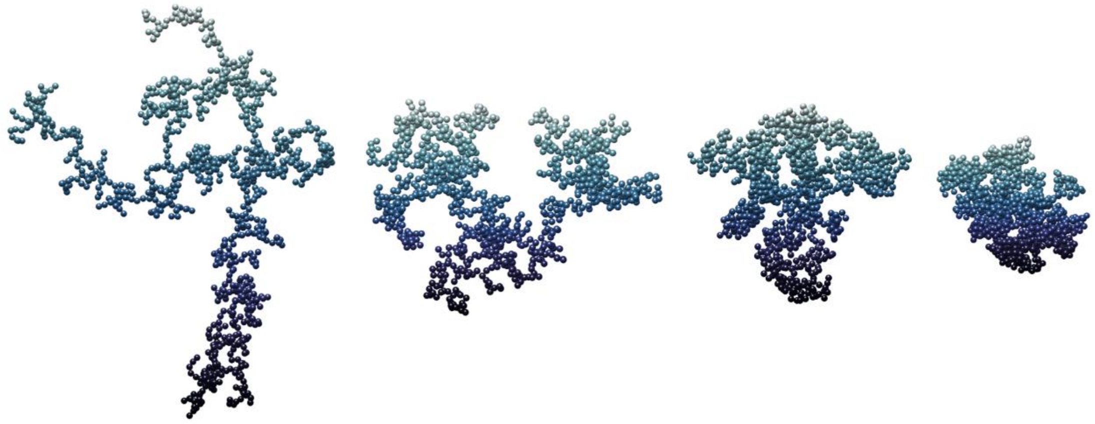

Supplementary Figure 3. Illustration of fractal aggregate morphology. Aggregates are shown with fractal dimensions (D_f) of 1.8, 2.0, 2.2 and 2.4 (from left to right). The aggregates contain 1000 monomers each.

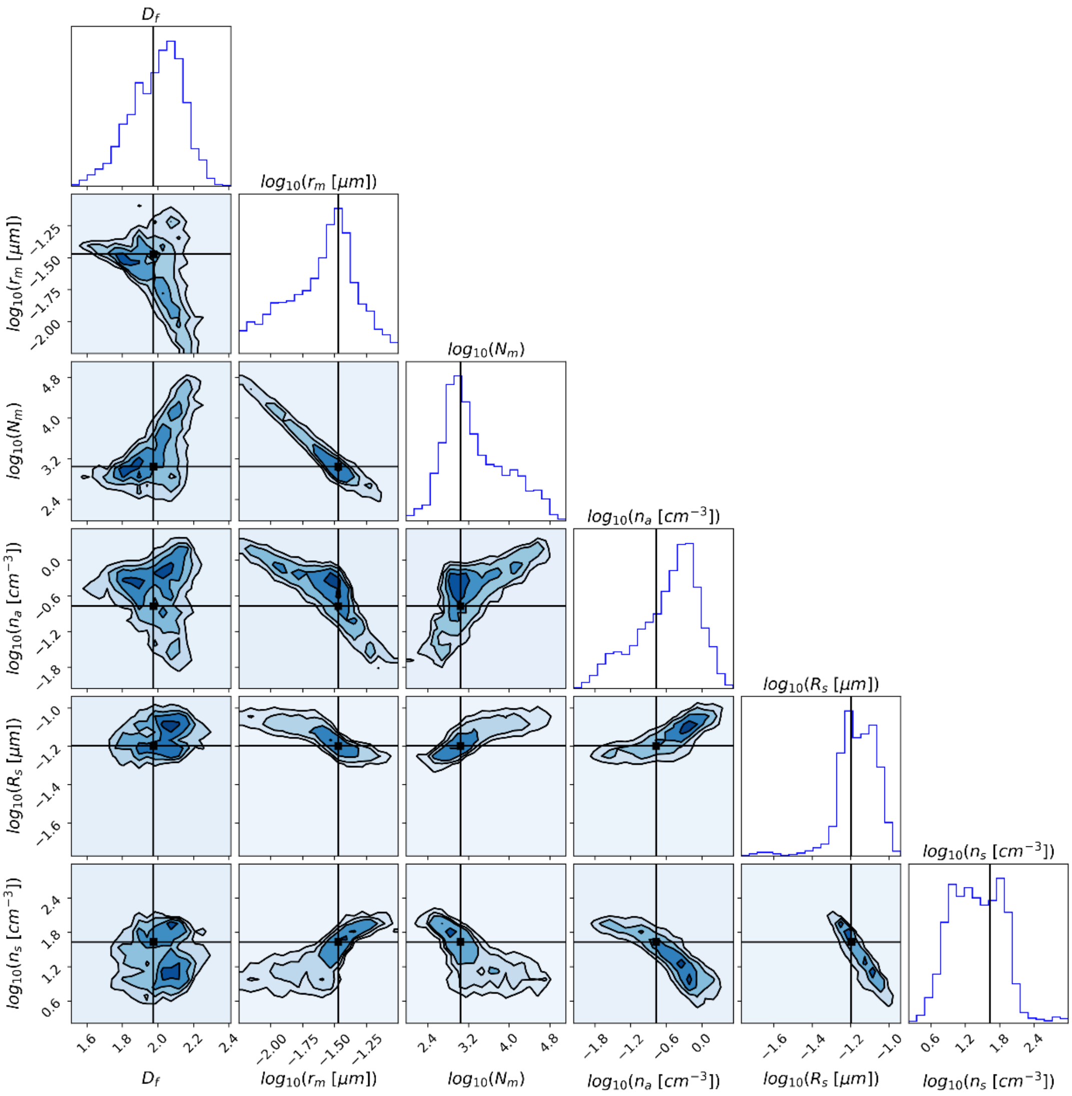

Supplementary Figure 4. Example of retrieval results. Retrieved probability density functions (PDFs) of the six free parameters and their joint distributions at 22.5km are shown for the scenario of the bimodal distribution of aggregates and spheres. The best fit values are denoted as solid black lines.

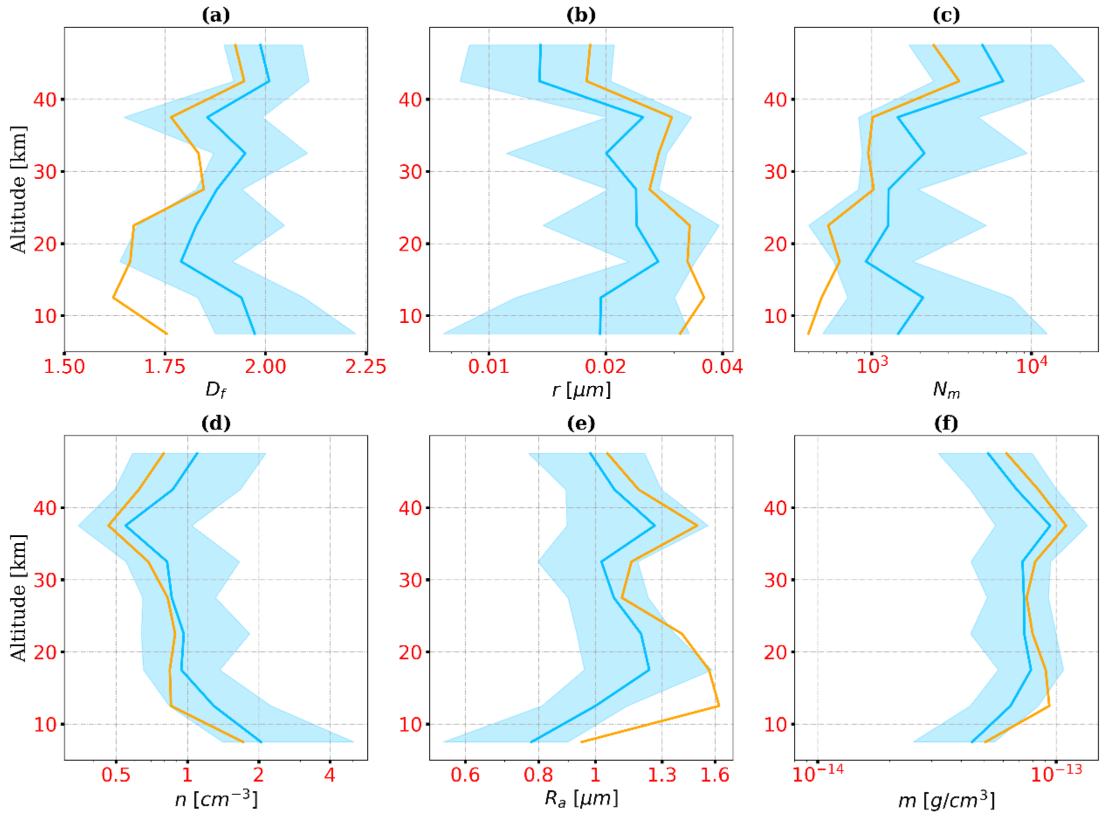

Supplementary Figure 5. Retrieved profiles of haze parameters. Same as Figure 3, but for the scenario of the monodispersed fractal aggregates constrained using all observations (orange) and forward scattering only (blue).

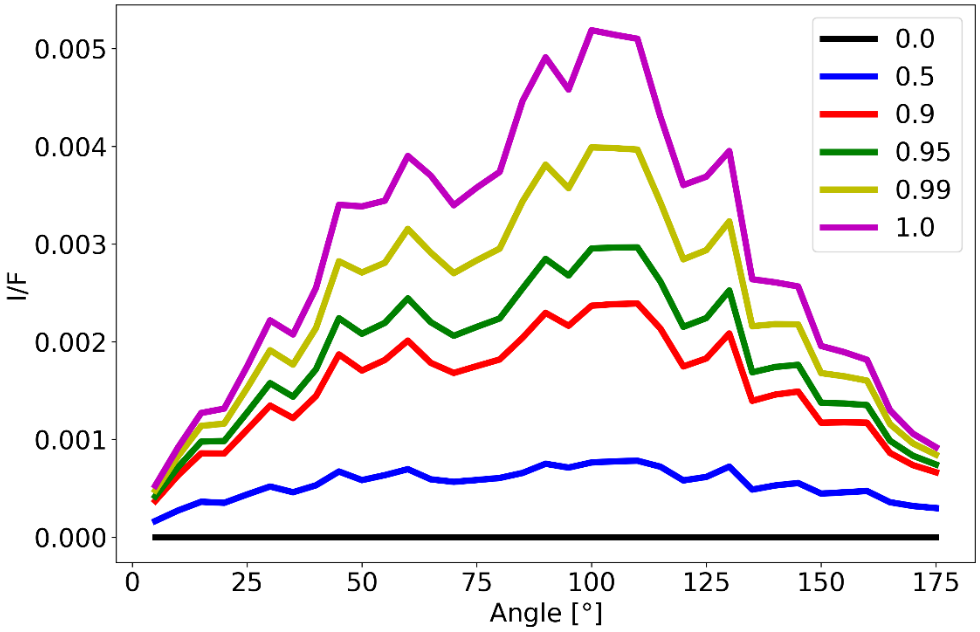

Supplementary Figure 6. I/F of secondary scattering. The scattering intensity by Pluto's surface and then by haze particles is integrated along the line-of-sight (LOS) at an altitude of 7.5km under assumptions of surface material single scattering albedos of 0.0 (black), 0.5 (blue), 0.9 (red), 0.95 (green), 0.99 (yellow), and 1.0 (magenta).

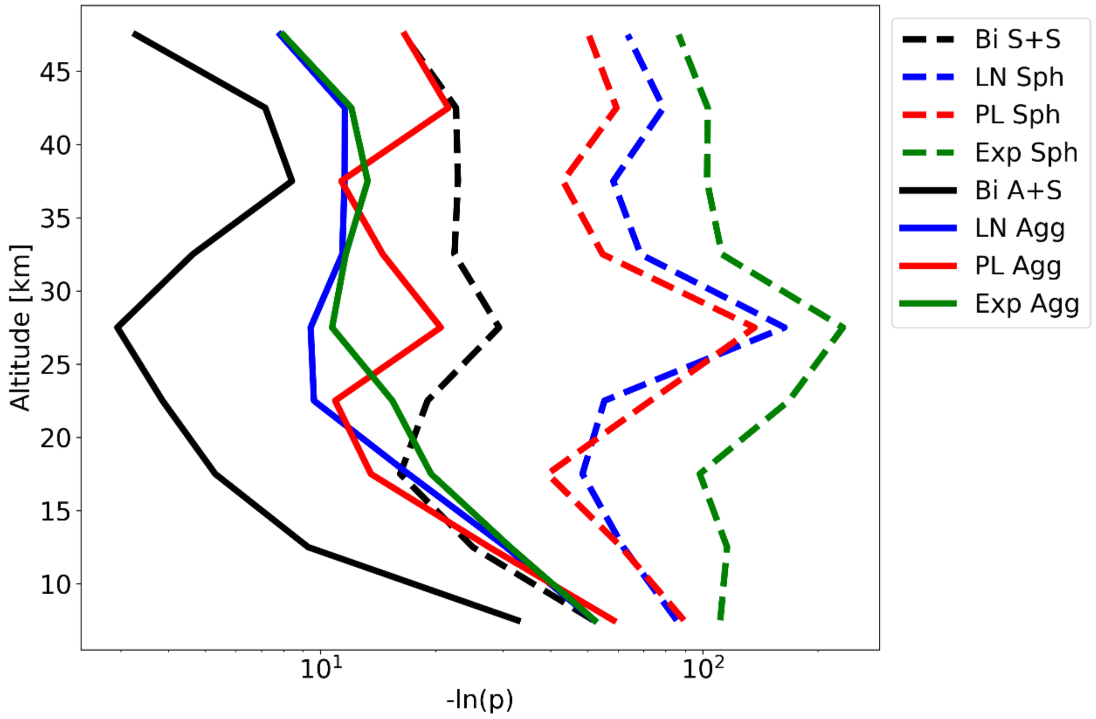

Supplementary Figure 7. Comparison of the goodness of fit of the scenarios. The goodness of fit is quantified by the negative of the natural logarithm of the posterior probability shown in Equation (20). The scenarios considered are: bimodal distribution of spheres (black dashed line), log-normal distribution of spheres (blue dashed line), power-law distribution of spheres (red dashed line), exponential distribution of spheres (green dashed line), bimodal distribution of aggregates and spheres (preferred scenario, black solid line), log-normal distribution of aggregates (blue solid line), power-law distribution of aggregates (red solid line), and exponential distribution of aggregates (green solid line).

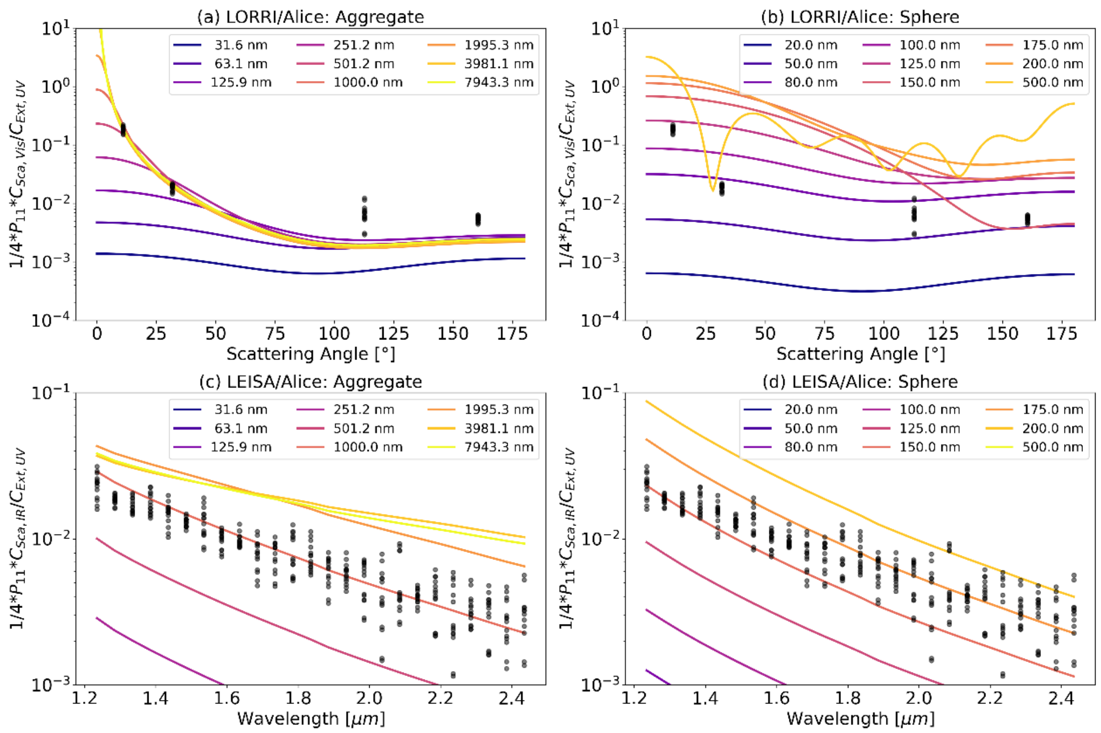

Supplementary Figure 8. Ratio of haze optical properties at different wavelengths. (a) Simulated ratio of local scattering intensity as a function of phase angles at 0.608μm to UV extinction at 0.185μm of two-dimensional aggregates (color curves) compared to the observed ratio of LORRI and Alice data at altitudes of 5-50km (black dots). (b) Same as (a) but for three-dimensional spheres. (c) Simulated ratio of the local scattering intensity spectrum at wavelengths of 1.235-2.435μm at a phase angle of 169.0° to UV extinction at 0.185μm (color curves) compared to the observed ratio of the LEISA spectrum to the Alice data (black dots). (d) Same as (c) but for three-dimensional spheres.

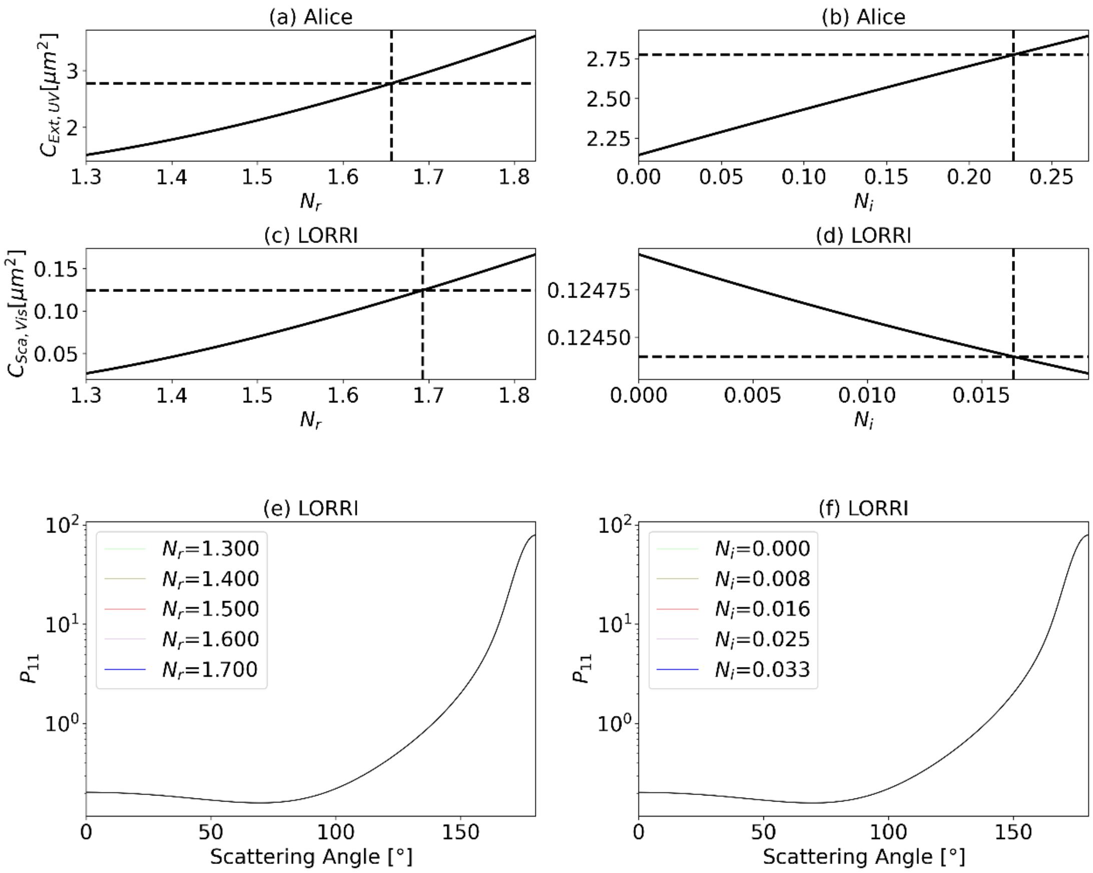

Supplementary Figure 9. Optical properties of aggregate haze particles with different refractive indices. (a) UV (0.185μm) extinction cross section of haze particles with a varying real (N_r) refractive index and fixed imaginary (N_i) refractive index. The particles are assumed to be two-dimensional 1μm aggregates consisting of 20nm monomers. The imaginary refractive index is fixed to that of "tholins"[38]. The real refractive index of "tholin" and corresponding UV cross section, which are the same as those used in the retrieval, are denoted as the vertical and horizontal dashed line, respectively. (b) Same as (a), but with a varying imaginary refractive index and fixed real refractive index. (c), (d) same as (a) and (b) but for the visible (0.608μm) scattering cross section, respectively. (e) Scattering phase functions of haze particles with a varying real refractive index and fixed imaginary refractive index. The curves overlap each other. (f) Same as (e), but for a varying imaginary refractive index and fixed real refractive index.

Supplementary Notes

Seven comma-separated-value (csv) files are included as a zip file in the supplementary information. They contain the processed observations, the retrieved haze morphology parameters, and corresponding scattering properties of haze particles.

"Observations.csv" contains the processed observations obtained by instruments onboard New Horizons shown in Figure 1 and 2, and also summarized in Table 1. The columns are as follows:
1. Altitude in km.
2-3. Extinction coefficients at 0.185μm with units of cm^{-1} obtained by Alice during ingress and egress, respectively.
4-7. Scattering coefficients at 0.608μm and at four phase angles (19.5°, 67.3°, 148.3°, and 169.0°) with units of cm^{-1} obtained by LORRI.
8-32. Scattering coefficients at 1.235-2.435μm with an interval of 0.05μm and at a phase angle of 169.0° with units of cm^{-1} obtained by LEISA.
33-44. Scattering coefficients at four wavelengths (0.624μm, 0.492μm, 0.861μm, and 0.883μm) and at three phase angles (18.2°, 38.8°, and 169.4°) with units of cm^{-1} obtained by MVIC.
45-87. Absolute uncertainties of the values shown in columns 2-44, respectively, with the same units of cm^{-1}.

"Morphology.csv" contains the retrieval results of the bimodally distributed haze particles. Six parameters are used to describe the morphology, which are shown in Figures 3a-3d. The columns are as follows:
1. Altitude in km.
2. Best fit values of the fractal dimension (D_f) of the aggregates.
3. The median of the Gaussian function fit to the PDF of D_f.
4. The width (1-σ uncertainty) of the Gaussian function fit to the PDF of D_f.
5-7. Same as columns 2-4, but for the values of the 10-based logarithm of the radius of the aggregate monomers (r_m) with units of μm.
8-10. Same as columns 2-4, but for the values of the 10-based logarithm of the number of monomers (N_m) in each aggregate.
11-13. Same as columns 2-4, but for the values of the 10-based logarithm of the aggregate number density (n_a) with units of cm^{-3}.
14-16. Same as columns 2-4, but for the values of the 10-based logarithm of the radius of the spherical particles (R_s) with units of μm.
17-19. Same as columns 2-4, but for the values of the 10-based logarithm of the sphere number density (n_S) with units of cm^{-3}.

"Mono_Both.csv" contains the scattering properties of monodispersed aggregates constrained using all observations, which are described in scenario (1) in Section 6.1 of Methods and shown as dashed lines with crosses in Figure 1. The columns are as follows:
1. Altitude in km.

2. Extinction coefficients at 0.185μm with units of cm^{-1}.
3-6. Scattering coefficients at 0.608μm and at four phase angles (19.5°, 67.3°, 148.3°, and 169.0°) with units of cm^{-1}.
7-31. Scattering coefficients at 1.235-2.435μm with an interval of 0.05μm and at a phase angle of 169.0° with units of cm^{-1}.
32-43. Scattering coefficients at four wavelengths (0.624μm, 0.492μm, 0.861μm, and 0.883μm) and at three phase angles (18.2°, 38.8°, and 169.4°) with units of cm^{-1}.

"Mono_Forward.csv" is the same as "Mono_Both.csv", but for monodispersed aggregates constrained using all observations except for the backscattering LORRI and MVIC data, which are described in scenario (2) in Section 6.2 of Methods and shown as dotted lines with squares in Figure 1.

"Bimod.csv" contains the scattering properties of the bimodally distributed haze particles which are described in scenario (3) in Section 6.3 of Methods and shown as solid lines with error bars in Figure 1. The columns are as follows:
1-43. Same as those in "Mono_both.csv".
44-85. Same as columns 2-43, but for the median of the Gaussian function fit to the PDFs of scattering properties.
86-127. Same as columns 2-43, but for the width (1-σ uncertainty) of the Gaussian function fit to the PDFs of scattering properties.

"Contribution_Agg.csv" contains the contribution of the aggregates to the scattering properties under the bimodal distribution scenario, which is shown in the blue shaded areas in Figure 2. The columns are as follows:
1. Altitude in km.
2. Extinction coefficients at 0.185μm with units of cm^{-1}.
3-183. Scattering coefficients at 0.608μm and at phase angles 0°-180° with an interval of 1° with units of cm^{-1}.

"Contribution_Sph.csv" is the same as "Contribution_Agg.csv", but for spheres, which is shown in the orange shaded areas in Figure 2.
